# Effects of Electrical Stimulation on Facial Paralysis Recovery after Facial Nerve Injury: A Review on Preclinical and Clinical Studies

**DOI:** 10.3390/jcm12124133

**Published:** 2023-06-19

**Authors:** Myung Chul Yoo, Jeong Hee Kim, Yong Jun Kim, Junyang Jung, Sung Soo Kim, Sang Hoon Kim, Seung Geun Yeo

**Affiliations:** 1 Department of Physical Medicine & Rehabilitation, College of Medicine, Kyung Hee University, Seoul 02447, Republic of Korea; 2 Department of Biomedical Science, Graduate School, Kyung Hee University, Seoul 02447, Republic of Korea; 3 Department of Pathology, College of Medicine, Kyung Hee University, Seoul 02447, Republic of Korea; 4 Department of Anatomy and Neurobiology, College of Medicine, Kyung Hee University, Seoul 02447, Republic of Korea; 5 Department of Biochemistry and Molecular Biology, College of Medicine, Kyung Hee University, Seoul 02447, Republic of Korea; 6 Department of Otorhinolaryngology Head & Neck Surgery, College of Medicine, Kyung Hee University, Seoul 02447, Republic of Korea

**Keywords:** facial nerve, regeneration, electrical stimulation, recovery

## Abstract

Various methods have been used to improve function and manage facial nerve injury. Although electrical stimulation therapy is frequently used to treat facial paralysis, its effects have been found to vary and no clear standards have been developed. The current review describes the results of preclinical and clinical studies evaluating the effectiveness of electrical stimulation therapy in promoting the recovery of a peripheral facial nerve injury. Evidence is presented showing the efficacy of electrical stimulation in promoting nerve regeneration after peripheral nerve injuries in both animal models and human patients. The ability of electrical stimulation to promote the recovery of facial paralysis was found to depend on the type of injury (compression or transection), the species of animal tested, the type of disease, the frequency and method of electrical stimulation, and the duration of the follow-up. Electrical stimulation, however, can also have potential negative outcomes, such as reinforcing synkinesis, including mistargeted axonal regrowth via inappropriate routes; excessive collateral axonal branching at the lesion site; and multiple innervations at neuromuscular junctions. Because of the inconsistencies among studies and the low quality of evidence, electrical stimulation therapy is not currently regarded as a primary treatment of facial paralysis in patients. However, understanding the effects of electrical stimulation, as determined in preclinical and clinical studies, is important for the potential validity of future research on electrical stimulation.

## 1. Introduction

The facial nerve is a mixed nerve that performs both motor and sensory functions. It is also mixed in the sense that it is a special sensory nerve that perceives taste in the anterior two-thirds of the tongue and is also a general sensory nerve responsible for the deep perception of the auricle, posterior wall of the external auditory meatus, ear lobe, and facial soft tissues [1,2]. Thus, the facial nerve consists of two efferent nerves and two afferent nerves and has four functions. Although facial nerve palsy is not a life-threatening condition, the resulting facial asymmetry affects interpersonal relationships and imposes significant psychological and neurological stress on the patient [3]. The considerable socio-economic costs associated with the treatment and rehabilitation of facial paralysis underscore the importance of identifying the cause of facial paralysis and providing the best curative treatment possible. To date, it has not been possible to achieve a complete recovery following a nerve cut or severe facial nerve damage resulting from a disease or accident, regardless of which treatment approach is used. In addition to changes in facial appearance and attendant psycho-social impacts, severe facial nerve injury and transection of the facial nerve can cause damage to the cornea because of the inability to close the eyes; it may make it difficult to retain water and food in the mouth. The impact of facial nerve palsy can extend beyond just limitations in facial muscle movement. For example, patients are frequently concerned about their ability to maintain oral competence, defined as the ability of the orbicularis oris and other perioral muscles to create sufficient pressure between the lips for various functions, such as speech, swallowing, smiling, and other facial expressions. The loss of oral competence can significantly affect a patient’s nutrition, hydration, social interactions, and mental well-being [4,5]. In addition, severe facial paralysis can lead to corneal lesions and eyelid malposition due not only to the loss of oral competence but also to the induced blink dysfunction caused by the weakness of the orbicularis oculi [6].

The facial nerve is the most susceptible to damage among cranial nerves owing to its long intracranial anatomical path and superficial extracranial location. Idiopathic facial palsy accounts for 75% of all cases, with trauma and Herpes Zoster Oticus being the next most common causes [7,8]. Other causes of facial nerve injury include traffic accidents, explosions, firearms, pressure from tumor growth, and bacterial or viral infection [9]. Facial paralysis may also be caused by cholesteatoma, acoustic schwannoma (vestibular schwannoma), or facial nerve schwannoma (facial neurinoma). In such cases, surgical intervention to cut the facial nerve is the only option. Therefore, in the case of irreversible damage to the facial nerve and muscle, the prognosis is often poor, even if the surgery successfully removed the tumor [10,11].

Physical therapy is a crucial component of the treatment of facial nerve injury, along with pharmacology and surgery, aiming to improve function [12]. Physical therapies, including exercise, biofeedback, laser treatment, electrotherapy, massage, and thermotherapy, are employed to accelerate the healing process, enhance facial function, and reduce potential complications [13]. However, the effectiveness of electrical stimulation remains inconclusive and further research is needed to determine its efficacy and potential risks. The 2013 guidelines from the American Academy of Otolaryngology-Head and Neck Surgery Foundation also stated that the lack of standardized therapy modalities and protocols makes it difficult to make recommendations regarding the use of physical therapy for patients with Bell’s palsy [14]. Additionally, a meta-analysis of studies conducted on humans also showed that electrical stimulation of the facial nerve was not effective in the early stages of facial palsy [12]. However, despite these challenges, electrical stimulation continues to be used as a therapeutic approach to enhance facial function and reduce potential complications. The primary goal of electrical stimulation remains to promote nerve regeneration and preserve muscle mass and contractile properties [15,16,17].

Researchers have performed preclinical studies involving rats to investigate the effects of electrical stimulation of nerves, including peripheral nerves, and muscle targets. These studies were guided by the hypothesis that such stimulation can have a favorable influence on axonal expansion and molecular events that govern the target muscle [18]. Electrical stimulation administered transcutaneously delivers a low-amplitude, pulsed electrical current that generally activates motor nerves innervating weak muscles, producing contractions, and preventing the development of muscle atrophy [19,20]. Monophasic electrical currents may also promote recovery from nerve injury by promoting tissue and nerve healing [18,21,22]. However, the effectiveness of electrical stimulation in Bell’s palsy remains unclear owing to the paucity of high-quality studies. Clinicians differ regarding the use of electrical stimulation for facial paralysis [23], with some asserting that electrical stimulation improves recovery while others are concerned about the potential adverse effects and increased risks of synkinesis.

If the clinical outcomes of electrical stimulation in relation to facial paralysis are not favorable and have limitations, it would be beneficial to examine preclinical results and compare them with clinical results to understand the differences. Previous studies on electrical stimulation related to facial paralysis have not comprehensively reviewed both preclinical and clinical research. Therefore, in this study, we aim to investigate the potential efficacy of electrical stimulation in the treatment of facial nerve injuries by including both preclinical and clinical evidence. By comparing preclinical research findings with clinical outcomes, we can gain a better understanding of the differences and provide valuable insights into the effectiveness of electrical stimulation for facial paralysis. To the best of our knowledge, no previous study has conducted a combined review of preclinical and clinical research on electrical stimulation in relation to facial nerve injury. Hence, this review aims to bridge this gap and contribute to the existing literature by evaluating the potential benefits of electrical stimulation across both preclinical and clinical domains.

## 2. Methods

The current review included preclinical studies in animals and clinical trials in humans involving facial nerve injury and electrical stimulation. Studies examining recovery from facial nerve injury and the effects of electrical stimulation, published between January 1999 and September 2022, were retrieved from three electronic databases—PubMed, SCOPUS, and EMBASE—by one of the authors (M.C.Y) using the search terms, ‘facial nerve’, ‘facial paralysis’, ‘recovery’, ‘Bell’s palsy’, ‘electrical stimulation’, and ‘nerve regeneration’. Eligible studies were independently screened and the relevant data were extracted. If there was any uncertainty about whether to include a study, a second investigator (S.G.Y.) acted as an arbitrator and made the final determination. Studies were excluded if they were: (a) unrelated to the topic and could not compare the effects of electrical stimulation alone; (b) unpublished data; (c) duplicates of previously published research; (d) published in a language other than English; and (e) pilot studies.

## 3. Results

**Figure 1:**
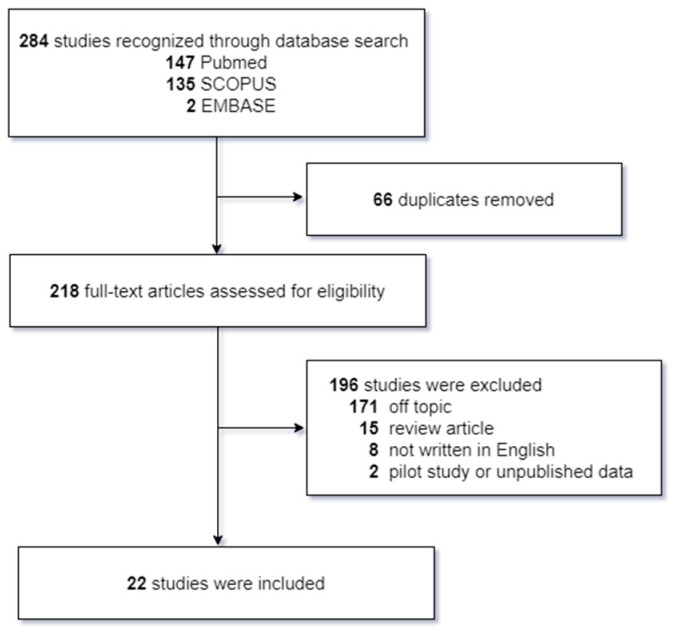
Flowchart of the literature review.

A total of 218 articles were obtained from the three databases using the specified search terms. However, only 22 studies (9.9%) met the inclusion criteria and were included in the analysis, as illustrated in Figure 1. The characteristics of these included studies are presented in Table 1 and Table 2.

### 3.1. Animal Studies of Electrical Stimulation (Table 1)

#### 3.1.1. Animal Studies Reporting Effective Electrical Stimulation Results

Charge-balanced transcutaneous electrical nerve stimulation (cb-TENS), performed after facial nerve injection in Sprague-Dawley (SD) rats, was reported to achieve functional recovery of the facial nerve [24]. In these studies, a 2.5-cm incision was made to expose the facial nerve trunk at the front of the ear in male SD rats. The main trunk of the left facial nerve was then surgically excised 10 mm distal to the stylomastoid foramen and the nerve was immediately anastomosed with a single 9-0 nylon outer membrane suture. Rats were divided into three groups: control, electrical stimulation at 20 Hz, and electrical stimulation at 40 Hz groups. The control group (22 animals) underwent surgery but did not receive cb-TENS treatment. Whisker movement improved over time in all groups, including unstimulated controls. However, recovery tended to be faster and more complete in groups treated with cb-TENS compared with the control group, with results in the 40 Hz group exhibiting a significant difference relative to the controls on the seventh post-surgery day (*p* < 0.0125). Moreover, molecular analyses showed that levels of the pro-inflammatory cytokines, IL-1β and IL-6, were significantly higher in the 20- and 40-Hz groups than in the control group (*p* < 0.015). Collectively, these observations indicate that Cb-TENS promotes and accelerates the recovery of the facial nerve in a rat model, greatly reducing the time for the recovery of whisker movement [24].

In another study, Foecking et al. sought to investigate whether electrical stimulation immediately after a facial nerve crush injury could further reduce the time to fully recover from facial paralysis [29]. In the experimental groups, electrical stimulation was given as 1-ms–wide square wave direct current stimulus pulses at 20 Hz (1.5 mA, 1 s), followed by a 1-s rest period. Electrical stimulation significantly reduced the time to the first recovery of the eye blink reflex (*p* < 0.001); among animals receiving one, two, or four sessions of electrical stimulation, full recovery of the blink reflex was achieved in an average of 12.5 days compared with 17.0 days in the control group (~26% reduction). The delivery of electrical stimulation only once was as effective as delivering electrical stimulation multiple times; all groups that received electrical stimulation recovered full vibrissae movement faster than untreated animals (*p* < 0.05), with a single, short (30 min) electrical stimulation session immediately after facial nerve crush injury significantly reducing the time to complete functional recovery. These results suggest that in cases where surgeons identify facial nerve damage during a surgical procedure, muscle reinnervation could be increased by a single session of short electrical stimulation administered to the nerve prior to wound closure [29].

In a study employing 24 female Wistar rats that received a transection injury or a crush injury of the facial nerve, electrical stimulation of an implantable stimulator demonstrated good therapeutic effects [27]. Four groups of six rats underwent the facial nerve injury procedure. Rats in Groups 1 and 2 suffered crush injuries to the main trunk of the nerve and those in Group 2 received brief electrical stimulation (BES) for an additional 1 h. Rats in Groups 3 and 4 received a transection injury to the main trunk and rats in Group 4 received BES for an additional 1 h. After nerve injury, the first wire was looped around the proximal stump of the facial nerve and the second wire was inserted into the muscle tissue next to the facial nerve at a position immediately adjacent to the first wire. The insulated wire was connected to an isobaric stimulator that delivered a 1.5-mA current with a pulse of 100 μs in a continuous 20 Hz train for 1 h. Crush injury animals that were administered BES showed significantly greater whisking amplitude 2, 4, and 6 weeks after treatment (*p* < 0.05). Collectively, these findings indicate that performing BES after facial nerve injury is associated with the acceleration of facial nerve function and improved regeneration of the facial nerve-specific pathway in a rat model [27].

#### 3.1.2. Animal Studies Reporting Ineffective Results of Electrical Stimulation

In some animal model studies, ineffective results of electrical stimulation for facial nerve injury were observed. Raslan et al. transected the right facial nerve in Wistar Unilever rats and electrically stimulated the proximal nerve cut for 60 min [34]. In the control animals, electrodes were immobilized on the nerve but no current was applied (sham stimulation). The operation was performed on the right facial nerve, with the application of electrical stimulation (*n* = 10) or sham stimulation (*n* = 9) during proximal nerve stump surgery. Motion analyses performed after facial injury showed poor vibrissal motion 4 weeks after sham stimulation. In the experimental group, whisking amplitude, velocity, and acceleration reached only 13–22% of that on the contralateral side at this same time point. This group showed a small improvement in the amplitude of agitation 8 weeks after injury; however, the velocity and acceleration were similar. For all parameters, values in the sham stimulation group were similar at 4 and 8 weeks after the injury. The authors of this study concluded that electrical stimulation may not be a universal treatment for facial nerve injury [34].

### 3.2. Human Studies of Electrical Stimulation (Table 2)

#### 3.2.1. Human Studies Showing Effective Electrical Stimulation Results

There have been few large prospective randomized studies of electrical stimulation as a treatment method related to the recovery of facial palsy in humans. Kim et al. conducted a prospective randomized study involving a relatively large number (*n* = 60) of patients with mild to moderate Bell’s palsy (House-Brackmann grade ≤ 4, Sunnybrook grade ≥ 40) to evaluate the effect of electrical stimulation on the resolution of facial palsy [17]. Thirty patients were treated with prednisolone and/or acyclovir and electrical stimulation within 7 days of symptom onset. Another 30 patients that were treated with only prednisolone and/or acyclovir were used as controls. The follow-up period was 1 year. Electrical stimulation was delivered with an electrical stimulator, which generated pulses with the following properties: average strength, 1.4 mA (sub-threshold); duration, 10 ms; interval, 50 ms; voltage, 10–20 V; frequency, 20 Hz with rectangular, monophasic spikes. All patients, except for one in the experimental group, were cured within 3 months. By contrast, five patients in the control group had not recovered normal facial function 6 months after the onset of paralysis. The facial function had fully recovered within 10.7 ± 5.7 weeks after the onset of facial paralysis in the experimental group compared with 13.4 ± 6.0 weeks in the control group. The experimental group showed an improvement in facial performance during the first 2 weeks and achieved complete recovery in a maximum of 10 weeks compared with 12 weeks for members of the control group, a difference that was significantly shorter (*p* < 0.05).

One year after the onset of facial paralysis, one patient in the experimental group showed synkinesis (oral-ocular synkinesis) accompanied by involuntary eye closure resulting from voluntary mouth movement, as well as static and dynamic facial asymmetry attributable to hypertonia, an unwanted secondary effect. Three patients in the control group showed an incomplete recovery of their facial function, all of whom also exhibited synchrony owing to secondary catatonia following facial paralysis. Sub-threshold continuous low-frequency electrical stimulation (SCLES) applied to the extratemporal part of the facial nerve immediately before the end of Wallerian degeneration resulted in a faster recovery of facial function and minimal facial sequelae after Bell’s palsy, suggesting SCLES as a new treatment approach for accelerating nerve regeneration and improving functional recovery after injury [17].

In another study on Bell’s palsy patients, in this case, a prospective study performed by Frigerio et al. [37], between 6 and 60 days in 40 patients with acute unilateral facial paralysis (House-Brackmann grades 4–6), electrical stimulation showed good therapeutic effects. Complete eye closure occurred in 55% of patients. In these individuals, initial eye twitching was observed at an average current of 4.6 ± 1.7 mA (average pulse width, 0.7 ms; frequency, 100–150 Hz) and complete eye closure was produced at an average stimulation strength of 7.2 ± 2.6 mA. Another study further revealed that the addition of 3 weeks of daily electrical stimulation shortly after the onset of facial palsy (4 weeks) improved functional facial movements [16]. Study participants were randomly divided into two equal groups: a conventional therapy group, treated with hot packs, facial expression exercises, and massages of the facial muscle and a group with conventional therapy plus concurrent electrical stimulation, performed 5 days per week for 3 weeks. In the control group, axonal degeneration was not detected in 16 patients (57.1%) but was found in 12 patients (42.9%). In the experimental group, 17 patients (53.1%) showed no evidence of axonal degeneration, whereas 15 (46.9%) exhibited axonal degeneration. The addition of daily electrical stimulation for 3 weeks within 4 weeks of the onset of facial paralysis improved measures of functional facial movement and electrophysiological outcomes in Bell’s palsy patients at a 3-month follow-up [16].

#### 3.2.2. Human Studies Showing Ineffective Electrical Stimulation Results

Some human studies have reported no effect of electrical stimulation, alone or in combination with conventional treatments, on facial nerve regeneration after facial nerve injury. In a prospective study investigating the therapeutic efficacy of electrical stimulation in Bell’s palsy patients, patients in both the control group and the experimental group were treated with a 5-min hot compress, a 10-min massage, and ten repetitions of exercise three times a week [15]. The experimental group additionally received electrical stimulation to the facial muscles for 30 min, delivered using a TENS device. The Facial Disability Index (FDI) improved, on average, by 52.8% (from 17.8% to 95.4%) in the control group and 49.8% (from 14.8% to 126%) in the experimental group, a difference that was not statistically significant [15].

In a final study, electrical stimulation was attempted in six patients with sublingual-facial-jump anastomosis (HFJA) [41]. While waiting for reinnervation after HFJA, patients in the hospital practiced placing electrical stimulation electrodes with the help of a mirror and then were advised to perform home training twice a day for 10 min, 5 days per week. Six patients (three men and three women) received electrical stimulation and thirty-three patients (seventeen men and sixteen women) did not. No differences in the innervation time after facial nerve reconstruction were seen between patients with and without electrical stimulation. Moreover, no significant differences in eFACE scores for exercise and resting symmetry were found between electrical stimulation and no electrical stimulation in comparisons of absolute differences between healthy faces and the two groups. The authors concluded that there was no evidence that electrical stimulation prevented or delayed reinnervation or increased synthetic locomotion in facial paralysis patients [41].

## 4. Discussion

Bell’s palsy is the most frequent form of facial paralysis encountered in clinical practice. Although around 70–80% of adults experience spontaneous recovery without treatment, some individuals may experience residual deficits [42,43]. These complications can include permanent facial paralysis, synkinesis (abnormal involuntary movements), muscle contracture, and facial asymmetry. Thus, patients with Bell’s palsy are greatly concerned about its resolution and the risk of developing permanent paralysis.

Bell’s palsy has traditionally been managed through a combination of physical and medical treatments. Physical treatment options have included massages, facial exercises, biofeedback, and electrical stimulation [13,44]. Although the effectiveness of the electrical stimulation for Bell’s palsy is unclear, it is thought to have the potential to improve recovery in patients with poor prognostic factors. Electrical stimulation may enhance muscle and nerve function, expedite the healing process, and reduce the risk of long-term paralysis in patients with acute Bell’s palsy [40]. Therefore, electrical stimulation is being explored as a physical therapy approach to improve this chronic condition; numerous preclinical and clinical studies have been performed to assess its effectiveness. For example, the continuous electrical stimulation of patients with chronic facial palsy for 6 months was reported to improve motor conduction latencies and clinical residuals, suggesting that prolonged electrical stimulation may facilitate reinnervation, potentially originating from neighboring healthy nerves, such as the fifth cranial nerve [38]. In addition, electrical stimulation-induced neuromodulation has been found to stimulate the remaining nerves, activating their inherent plasticity, which may lead to sensory and motor reorganization [45]. Continuous electrical afferent stimulation at a sub-sensory level has been reported to trigger a neurophysiological mechanism that can be activated by externally controlled sensory input, thereby inducing modulatory effects within the sensorimotor cortex [46].

Several studies, however, have reported insufficient evidence to adequately support the effectiveness of electrical stimulation in patients with facial paralysis. A 2011 Cochrane review on physical therapy for facial paralysis mentioned various treatment modalities, including exercise, biofeedback, laser treatment, and electrical therapy; however, it reported a lack of sufficient evidence to support their effectiveness [13]. Furthermore, contractions generated by electrical stimulation can further activate already overactive muscles and reinforce abnormal patterns [47,48]. Therefore, further research is needed to better understand the potential benefits and limitations of these treatment approaches.

Basic research experiments, coupled with the histopathological analysis of tissue samples, can enhance our understanding of the various mechanisms underlying the effects of electrical stimulation and provide valuable insights for improving patient prognosis (Table 1). Most preclinical studies in animal models tested the effectiveness of electrical stimulation by invasive insertion or wrapping electrodes. Electrical stimulation in rat models of crushed facial nerves has been shown to enhance the expression of regeneration-associated genes and proteins, such as brain-derived neurotrophic factor (BDNF), α1-tubulin, and growth-associated protein-43 (GAP-43) [31]. Electrical stimulation was also found to enhance the expression of cytokines and/or the release of neurotrophins, which play crucial roles in peripheral nerve regeneration and functional recovery [24,49]. Although several methods of stimulation, involving direct invasive insertion or implantation of electrodes, have shown efficacy in preclinical studies, the clinical applications of these methods are limited. Many preclinical studies have utilized surgically implanted stimulation devices, such as two Teflon-coated wires, which have limited applications in humans. Thus, the types, frequencies, and duration of electrical stimulation, as well as the quantitative methods of assessing recovery and the types of injury, have varied widely in preclinical studies, leading to heterogeneous results.

In most clinical studies, electrical stimulations were delivered transcutaneously (Table 2). Furthermore, the types of conditions varied among these studies, including Bell’s palsy, acoustic neuroma excision, and hypoglossal-facial-jump anastomosis. Six of the studies tested found that electrical stimulation yielded positive results, whereas three studies found that electrical stimulation yielded no significant benefits. Most studies utilized electrical stimulations delivered at intensities sufficient to induce muscle contractions, with the exception of one study that used sub-sensory level stimulations [17]. Unfortunately, reporting on the adverse effects was inadequate or nonexistent. For future research, it is necessary to establish standardized protocols for electrical stimulation parameters, treatment duration, and evaluation methods. This standardization will enable meaningful comparisons among studies and facilitate meta-analyses to analyze the results. Furthermore, the potential adverse effects of synkinesis resulting from electrical stimulation should be considered in studies of patients with acute facial paralysis. It is crucial to investigate whether these adverse effects are exacerbated or alleviated by electrical stimulation. Additionally, studies are needed to determine the optimal duration of electrical stimulation and whether, and at what point, it should be continued or discontinued.

This review provides an overview of preclinical studies conducted primarily in rodent models and human research findings. Rodent models, commonly used in preclinical studies, have advantages, such as small size and suitability for experimental nerve repair. However, data obtained from these models should be critically evaluated. Interpretation of the results from preclinical and clinical studies should take into consideration the excellent regenerative capacity and smaller limb length observed in small animal models, particularly in mice [50]. Selecting a consistent and appropriate time point for evaluating facial nerve regeneration and functional recovery in mouse experiments is of utmost importance. This is particularly crucial in order to prevent data distortion and bias, ensuring the reliability of the results [50]. Additionally, aligning the findings from mouse experiments with those obtained from larger animal models can provide further validation and strengthen the translational potential of basic research on nerve regeneration in clinical applications.

The application of electrical stimulation in all facial paralysis patients primarily focuses on addressing facial weakness and preventing further damage on the affected side. However, in the context of emerging technologies, there is ongoing research on methods where electrical stimulation is induced by the contralateral side of the face. This approach involves recording signals from healthy neural tissue to extract neural signals that are then used to treat the damaged side of the face. Implants equipped with flexible muscle stimulation electrodes and electromyographic recording arrays have been used, along with a portable pulse generator, to capture and stimulate symmetric facial movements [51]. There are various types of electrodes used for neural stimulation. These include extra-neural electrodes, such as cuff electrodes and flat interface nerve electrodes (FINE), as well as intraneural electrodes, such as longitudinal intra-fascicular electrodes (LIFE), transverse intra-fascicular multichannel electrodes (TIME), and multielectrode arrays. Furthermore, these technologies allow for the connection of individual wires to percutaneous connectors or implantable wireless interfaces, enabling recording or electrical stimulation of the facial nerves [52,53]. The advancement of bioelectrical interfaces and nanotechnology holds the potential to expand the possibilities for successful therapeutic interventions in future research. These advancements can contribute to restoring lost neural inputs and facilitating muscle function recovery.

## 5. Conclusions

Despite a long history of peripheral nerve electrode development, commercialization remains limited and standard surgical methods have yet to be established; although, data-assessing factors that contribute to the failure of different clinical applications continue to accumulate. The effect of electrical stimulation on the regeneration process after facial nerve injury depends on the cause of facial paralysis, the degree of damage, the method of electrical stimulation, the number of stimuli used, and the follow-up period; for animal studies, the method used to induce the injury and the type of animal are contributing factors. Reports on the ability of electrical stimulation to promote recovery from facial paralysis have yielded varying results; the current review indicates that the therapeutic efficacy of electrical stimulation differs between humans and animals. The effectiveness of electrical stimulation in patients with facial nerve damage caused by viral reactivation, trauma, and benign and malignant tumors, as well as the resulting facial paralysis, may differ from its effectiveness in preclinical studies in animals with artificial damage. However, the preclinical and clinical studies described in this review provide clues to the possible ranges of electrical stimulation for optimal facial nerve treatment. More detailed and objective future studies on the effects of electrical stimulation on facial nerve regeneration after facial nerve injury are needed to determine optimal facial nerve treatments.

## Figures and Tables

**Table 1:** Studies on animal facial nerve regeneration by electrical stimulation.

Author	Species and/or Sample	Injury Type	Stimulation Methods : Type/Duration/Location	Experimental Design	Results/Conclusion/Suggestions
**Effective results by electrical stimulation (ES)**					
Cho et al., 2022 [24]	Sprague-Dawley rats, male	Transection: main trunk of the left facial nerve was cut 10 mm and the nerve was immediately repaired	**Type**: charge-balanced transcutaneous electrical nerve stimulation (Cb-TENS) with a biphasic electric pulse, **Duration**: daily for seven days and then twice a week for 3 weeks (total 4 weeks) **Location**: skin patch electrode of the preauricular area	- Group 1: The control group with no TENS (*n* = 22). - Group 2: Injury with Cb-TENS at 20 Hz (*n* = 22). - Group 3: Injury with Cb-TENS at 40 Hz (*n* = 22). **Parameters:** Whisker movement Histopathological evaluation with Transmission electron microscopy (TEM) **Post-injury duration**: 28 days	Rats in the Cb-TENS groups showed faster and better recovery of whisker movement than those in the control group. The 40 Hz group showed significantly better movement at the first week after injury. Regeneration of the myelin sheath was remarkably rapid and thicker in the 20 Hz and 40 Hz groups than in the control group. Cb-TENS promoted and accelerated facial nerve recovery, as it significantly reduced the recovery time for whisker movement.
Brown MS et al., 2020 [25]	Wistar rats	Transection and neurorrhaphy at main trunk of the right facial nerve	**Type**: fine wire electrodes **Duration**: 100 μs charge-balanced square waves; peak-to-peak amplitude 3 V. 20 Hz train for 30 min **Location**: hooked around the nerve 2 mm proximal to the intended axotomy site	- Group 1: control (*n* = 3). - Group 2: neurorrhaphy only (*n* = 4). - Group 3: neurorrhaphy + ES (*n* = 8). - Group 4: neurorrhaphy + ES + polyethylene glycol (PEG) (*n* = 8). **Parameters:** Eye blink reflex Whisking movement Tissue analysis Retrograde tracing	Incorporating short-duration intraoperative electrical stimulation into neurorrhaphy in this animal model demonstrates potential positive neurological effects in the surgical treatment of facial nerve injury.
Deng et al., 2018 [26]	Sprague-Dawley rats, male	Transection: 1-cm defect surgically introduced on the right facial nerve.	**Type**: transcutaneous electrodes **Duration**: an electrical pulse of 3 V, 20 Hz and 0.3 mA for 1 h each day (total 28 days) **Location**: the orbicularis oris muscle of the right paralyzed face	- Group 1: main trunk of the right facial nerve transection only (*n* = 20) - Group 2: surgery + ES group (*n* = 20). - Group 3: normal control group (*n* = 20). **Parameters:** Facial muscle movement, compound muscle action potentials (CMAPs), Histological structure The expression levels of S100B and NF200. **Post-injury duration**: 28 days	The ES group showed a higher amplitude and shorter latency of compound muscle action potential from day 14 to 28 after surgery, as well as increased number of axons and expression of S100B and NF200 proteins. ES promotes outgrowth and myelination of axons and a partial functional recovery of facial muscles in rats with injured facial nerves.
Mendez et al., 2018 [27]	Wistar rats	Crush and transection After transection injury, the cut nerve ends were immediately repaired by direct end-to-end anastomosis.	**Type**: implantable stimulation device (two silver Teflon coated wires for BES) **Duration**: a 1.5 mA current in pulses of 100 microseconds in a continuous 20 Hz train for a period of 1 h only. **Location**: first wire was looped around the proximal stump of the facial nerve. The second wire was imbedded into muscle tissue adjacent to the facial nerve.	- Group 1 (*n* = 6): crush injury at the main trunk of the nerve - Group 2 (*n* = 6): crush injury at the main trunk of the nerve + BES (1 h) - Group 3 (*n* = 6): transection injury at the main trunk of the nerve - Group 4 (*n* = 6): transection injury at the main trunk of the nerve + BES (1 h) **Parameters:** Facial nerve functional outcome (2, 4, and 6 weeks): whisking movement Buccal and marginal mandibular branches of the facial nerve were each injected with different neurotracers at 3 months post-injury. **Post-injury duration**: 6 weeks	BES (brief electrical stimulation) demonstrated improved whisking movement and pathway-specific regeneration with BES following facial nerve injury. Performing BES after facial nerve injury is associated with accelerated facial nerve function and improved facial nerve specific pathway regeneration in a rat model.
Mendez et al., 2016 [28]	Wistar rats	Transection: right facial nerve transection and repair at the main trunk of the nerve.	**Type**: implantable stimulation device (two silver Teflon coated wires for BES) **Duration**: a 1.5 mA current in pulses of 100 microseconds in a continuous 20 Hz train for a period of 1 h only. **Location**: first wire was looped around the proximal stump of the facial nerve. The second wire was imbedded into muscle tissue adjacent to the facial nerve.	- Group 1 (*n* = 9): transection injury - Group 2 (*n* = 9): transection injury + BES **Parameters:** Facial nerve functional outcome (2, 4, and 6 weeks): whisking movement **Post-injury duration**: 6 weeks	In rats subjected to facial nerve transection and neurorrhaphy at the main trunk of the facial nerve, those that received BES after transection showed significantly accelerated whisker movement compared with those that did not.
Foecking et al., 2012 [29]	Sprague -Dawley rats, male	Crush	**Type**: implantable stimulation device **Duration**: 1 ms-wide square wave direct current stimulus pulses at 20 Hz (1.5 mA, 1 s), followed by a 1s rest period. Daily 30 min sessions of ES for 1, 2, 4, or 7 d. **Location**: negative electrode terminal was then sutured approximately 2 mm proximal to the crush injury site and the positive electrode terminal to connective tissue about 3 to 5 mm away from the negative terminal on the opposite side of the nerve	The animals were divided into six groups (*n* = 6 in each group) - Group 1: no ES - Group 2: one 30 min session of ES immediately following the injury (1 session); - Group 3: Group 2 + 1 d later (total 2 sessions) - Group 4: Group 2 + 1 d to 3 d later (total 4 sessions) - Group 5: Group 2 + 1 d to 6 d later (total 7 sessions) - Group 6: stimulated daily until complete recovery. **Parameters:** The eye blink reflex Vibrissae movement **Post-injury duration**: measured until complete recovery.	One session of ES was as effective as daily stimulation at enhancing the recovery of most functional parameters.
Kim et al., 2011 [30]	New Zealand white rabbits, male	Crush	**Type**: implantable stimulation device (two Teflon coated wires) **Duration**: Subthreshold continuous direct current ES with 20-Hz square-wave pulses for 4 weeks. **Location**: the proximal stump on the facial nerve	- Group 1: Crush injury + no ES - Group 2: Crush injury + 30 min session of ES (1 session only) **Parameters:** Electrophysiological test Histological study Functional recovery of vibrissae movement **Post-injury duration**: 30 days	Vibrissae movement returned significantly earlier on the ES side. Electrophysiologically, the stimulated side had a significantly shorter latency, longer duration, and faster conduction velocity. Light and transmission electron microscopy revealed that the electrical stimulation also markedly decreased Wallerian degeneration. Subthreshold, continuous, low-frequency ES immediately after a crush injury of the facial nerve results in earlier recovery of facial function and shorter overall recovery time.
Hadlock et al., 2010 [18]	Wistar rats	Transection: immediate microsurgical repair.	**Type**: a hooked pair of platinum stimulating electrodes **Duration**: suprathreshold stimulation of the facial nerve was delivered for 60 min using a 3-V, 20-Hz square-wave **Location**: main trunk of the facial nerve In the MEC and BES+MEC groups, animals received 5 min of daily massage to the left whisker pad	Sixty rats were randomized to three groups (*n* = 20 in each group): - Group 1: BES - Group 2: mechanical stimulation of the whisker pad (MEC) - Group 3: both in combination (BES+MEC) **Parameters:** Whisking behavior **Post-injury duration**: 13 weeks	Rats that received either BES or MEC of the whisker pad showed accelerated recovery of whisking behavior. A combination of both interventions did not show accelerated recovery.
Sharma et al., 2010 [31]	Sprague -Dawley rats, male	Crush	**Type**: implantable stimulation device (two Teflon coated wires) **Duration**: crush injury site at a frequency of 20 Hz for 30 min. **Location**: proximal to the crush injury	The rats were randomized to eight groups (*n* = 4–8 in each group): - Group 1: control - Group 2: prednisone (P) only - Group 3: ES only - Group 4: ES + P - Group 5: systemic testosterone propionate (TP) only - Group 6: TP + P - Group 7: ES + TP - Group 8: ES + TP + P **Parameters:** The eye blink reflex Vibrissae orientation and movement **Post-injury duration**: 13 weeks	ES of the proximal nerve stump most effectively accelerated the initiation of functional recovery. ES or TP treatments enhanced recovery of some functional parameters more than P treatment. A combinatorial treatment strategy of using ES and TP together promises to be an effective therapeutic intervention for promoting regeneration following facial nerve injury.
Hetzler et al., 2008 [32]	Sprague -Dawley rats, male	Crush	**Type**: implantable stimulation device (two Teflon coated wires) **Duration**: post–crush injury day 1 and daily for 30 min until complete recovery Set to generate pulses every 2 msec for 5-msec duration **Location**: The right facial nerve, proximal to the crush injury	Rats were randomly assigned to four groups: - Group 1: control (*n* = 9) - Group 2: crush injury + ES (*n* = 8) - Group 3: crush injury + TP (*n* = 8) - Group 4: crush injury + ES + TP (*n* = 6) **Parameters:** Vibrissae orientation/movement Semi eye blink, and full eye blink. **Post-injury duration**: measured until complete recovery.	Complete recovery time was significantly reduced with concurrent ES and TP administration. The concurrent use of two existing treatment methods, electrical stimulation and gonadal steroid treatment, may potentially enhance nerve regeneration and expedite the time required for full functional recovery.
Lal et al., 2008 [33]	Sprague -Dawley rats, male	Crush	**Type**: implantable stimulation device (two Teflon coated wires) **Duration**: post–crush injury day 1 and daily for 30 min until complete recovery. Set to generate pulses every 2 msec for 5-msec duration **Location**: The right facial nerve, proximal to the crush injury	Rats were randomly assigned to two groups: - Group 1: crush injury + sham stimulation (*n* = 9) - Group 2: crush injury + ES (*n* = 8) **Parameters:** Eye blink reflex Vibrissae orientation Vibrissae movement. **Post-injury duration**: measured until complete recovery.	ES initiated and accelerated facial nerve recovery in the rat model.
**Non-effective results by ES**					
Raslan et al., 2019 [34]	Wistar Unilever rats, female	Transection of femoral and facial nerve The nerves were repaired by end-to-end suturing.	**Type**: implantable stimulation device (two Teflon coated wires) **Duration**: BES (1 h, 20 Hz) of the proximal nerve stump **Location**: - A stainless steel wire (50 μm thick) - both proximal femoral and facial nerve stump were electrically stimulated for 1 h (0.1 ms width, 20 Hz).	The animals were divided into four groups: - Group 1: sham-stimulated (SS) with femoral transection (*n* = 8) - Group 2: ES group with femoral nerve transection (*n* = 10) - Group 3: SS with facial nerve transection (*n* = 9) - Group 4: ES group with facial nerve transection (*n* = 10) **Parameters:** Video recordings were performed for gait analysis Whisker motion analysis **Post-injury duration**: 20 weeks	BES enhances sensory neuron reinnervation after femoral nerve injury, which may in turn, and in addition to direct effects on motor axon regeneration, promote restoration of motor function. However, BES did not improve the functional outcome of facial nerve injury as estimated by vibrissal motion analysis at 8 weeks after injury.
Sinis et al., 2009 [35]	Wistar rats	Transection	**Type**: acupuncture needle electrodes. **Duration**: The muscles were stimulated for 5 min by applying square 0.1 ms pulses at suprathreshold amplitudes (typically 3.0–5.0 V) ES of the vibrissal muscles 3 times a week over 2 months. **Location**: two acupuncture needle electrodes were inserted towards the levator labii superioris	The animals were divided into six groups (*n* = 16 in each group): - Group 1: control - Group 2: FFA only - Group 3: Resection - Group 4: FFA + SS - Group 5: FFA + ES - Group 6: FFA + mechanical stimulation **Parameters:** Video recordings were performed for vibrissal motor performance Degree of collateral axonal branching The number of motor endplates The quality of the reinnervation **Post-injury duration**: 2 months	ES did not improve functional outcome, but rather reduced the number of innervated motor endplates to approximately one-fifth of normal values and failed to reduce the proportion of poly-innervated motor endplates. ES is not beneficial for recovery of whisker function after facial nerve repair in rats.

**Abbreviations:** electrical stimulation, ES; brief electrical stimulation, BES; cb-TENS, charge-balanced transcutaneous electrical nerve stimulation; transmission electron microscopy, TEM; multichannel cuff electrodes, MCE; electromyography, EMG; testosterone propionate, TP; sham-stimulation, SS; facial-facial anastomosis: FFA; compound muscle action potentials (CMAPs).

**Table 2:** Human studies on facial nerve regeneration by electrical stimulation.

Author	Diseases/Etiology	Design	Stimulation Methods	Experimental Design	Results/Conclusion/Suggestions
**Effective results by electrical stimulation (ES)**					
Mäkelä et al., 2021 [36]	Bell’s palsy, Ramsay Hunt sndrome, and sequelae of temporomandibular joint replacement	15 patients with acute facial nerve palsy palsy.	**Type**: transcutaneous electrodes **Duration**: a biphasic square wave with symmetric positive and negative phases of equal width using phase duration of 0.4 ms and 250 Hz pulse **Location**: the zygomatic branch of the facial nerve. One electrode was attached just lateral to the orbital rim and the other at approximately 0.5 cm distance laterally.	Day 1: a two-hour TV watching session in which an electrically induced blink was delivered every 5 s. Day 2: TV watching session without electrically induced blinking. The two conditions were counterbalanced. **Parameters**: Subjective ocular symptoms (Dry Eye Questionnaire ) Visual acuity (Logarithm of the Minimum Angle of Resolution)	**Adverse event**: not specified The stimulation produced a blink in 8 participants (53%). Electrically elicited blink is a promising method for reducing the eye symptoms in individuals with acute facial nerve palsy
Kim et al., 2016 [17]	Bell’s palsy (mild-to-moderate grade)	Prospective randomized study	**Type**: transcutaneous electrodes **Duration**: Sub-threshold, continuous, low-frequency-impulse electrical stimulation (SCLES) at 20 Hz keep contining ES after 2 months from onset of facial palsy **Location**: The surface electrode was placed on the main branch of the facial nerve at the tragal pointer as a cathode, and on the intra-temporal area around the stylomastoid foramen as an anode	- Group 1: medical treatment only (*n* = 30) - Group 2: medical treatment + SCLES (*n* = 30) **Parameters**: Change of facial function (House-Brackmann and Sunnybrook scales)	**Adverse event**: Contact dermatitis (13.3%; 4/30) oral-ocular synkinesis (3.3%; 1/30) Facial function of Group 2 was regained completely within 10.73 ± 5.7 weeks after the onset of facial palsy, and the Group 1 recovered within 13.4 ± 6.0 weeks. The overall rate of patient recovery among those in Group 2 (96%) was significantly higher than the rate among those in Group 1 (*p* < 0.05). The drug regimen plus SCLES was more effective in treating Bell’s palsy than the conventional drug treatment alone.
Frigerio et al., 2015 [37]	Bell’s palsy, Lyme disease, Ramsay Hunt syndrome, temporal bone fracture, autoimmune disease Acute unilateral facial paralysis, House-Brackmann grades 4-6.	Prospective, double-blinded, randomized, placebo-controlled study.	**Type**: transcutaneous electrodes **Duration**: 0.4- to 1-ms pulse width, 100 to 150 Hz, and 1 to 15 mA. Bipolar, charge-alanced pulse **Location**: The cathode was positioned 1 cm lateral to the orbital rim. The anode was moved in an arc around the cathode, spanning 90 degrees (30 degrees above to 60 degrees below the cathode) in 10-degree increment	- Group 1: eye blink group (*n* = 16) by initial ES - Group 2: incomplete eye blink group (*n* = 12) by initial ES - Group 3: no motor response group (*n* = 4) by initial ES **Parameters**: Complete eye closure (Full blink) Synkinesis cutaneous sensation (Wong-Baker Faces Pain Rating Scale) Average current of initial eye twitch, complete closure	**Adverse event**: Synkinesis (34.3%; 11/32) Complete eye closure was achieved in 12 weeks later (*n* = 32) - Group 1: 75% (12/16) - Group 2: 25% (3/12) - Group 3: 50% (2/4) Complete eye closure was achieved in 12 months later (*n* = 20) - Group 1: 83% (5/6) - Group 2: 50% (5/10) - Group 3: 50% (2/4) Transcutaneous facial nerve stimulation may artificially elicit eye blink in a majority of patients with acute facial paralysis.
Tuncay et al., 2015 [16]	Bell’s palsy.	Prospective randomized study	**Type**: transcutaneous electrodes **Duration**: monophasic waveform 100 ms of pulse duration, 300 ms of interpulse interval, and a pulse rate of 2.5 Hz ES was performed 5 days per week for 3 weeks. **Location**: 11 facial muscles (frontalis, corrugator supercilii, palpebral part of orbicularis oculi, levator labii superioris alaeque nasi, levator labii superioris, levator anguli oris, risorius, orbicularis oris, depressor anguli oris, depressor labii inferioris, and levator menti)	- Group 1: control group, oral steroids + conventional therapy only (*n* = 28) - Group 2: oral steroids + conventional therapy + ES (*n* = 32) Conventional therapy: hot pack, facial expression exercises, and massage **Parameters**: House-Brackmann scale Facial disability index latency and amplitude of CMAPs	**Adverse event**: not specified The addition of 3 weeks of daily ES after facial palsy onset (4 weeks) improved functional facial movements and electrophysiologic outcome measures at 3-month follow-up in patients with Bell’s palsy.
Targan et al., 2000 [38]	Chronic facial nerve injury caused by Bell’s palsy, acoustic neuroma excision.	Case control study	**Type**: transcutaneous electrodes **Duration**: a monophasic current waveform, pulse duration of 86 μs, and delivered 1 pulse every 700 ms. **Location**: orbicularis oculi, frontalis, zygomatic, and nasalis were stimulated with submotor level intensity For 30 min daily during the first month, 1 h daily during the second month, and 2 h daily during the third month. for 6 h while sleeping at night during the fourth month. buccinator, mentalis, orbicularis oris, and mandibularis were stimulated for 30 min (fifth month) and 60 min (sixth month) daily.	- Group 1: Bell’s palsy (*n* = 12) - Group 2: acoustic neuroma excision (*n* = 5) **Parameters**: House-Brackmann score Motor nerve conduction latency Clinical residuals score	A 6-month program of electrical stimulation partially reverses chronic deficiencies in motor conduction latencies and clinical residuals and may prove beneficial to patients with chronic facial nerve paresis.
Gittins et al., 1999 [39]	Chronic, moderate to severe facial nerve palsy with acoustic neuroma resection, Ramsay Hunt syndrome.	Case control study	**Type**: transcutaneous electrodes **Duration**: a monophasic current waveform, pulse frequency and width were set to 10 Hz and 200 microsec **Location**: medial and lateral canthi	- Group 1: normal control (*n* = 30) - Group 2: chronic, moderate to severe facial nerve palsy (*n* = 10) **Parameters**: Voluntary and spontaneous eyelid movement and lid velocity	The use of transcutaneous electrical nerve stimulators over a period of three months results in a significant improvement in voluntary eyelid movements by reducing the stiffness of the eyelid mechanics.
**Non-effective results by ES**					
Shoman et al., 2022 [40]	Bell’s palsy	Prospective, randomized, single-blind, controlled study	**Type**: transcutaneous electrodes **Duration**: -LLLT: A total of 12 LLLT sessions (twice per week over six weeks). Each session lasted 8 min (1min/point) for 8 points. -ES: 0.1 ms to 1 ms and a frequency of 50 Hz. A total of 12 ES sessions (twice per week over six weeks). **Location**: - LLLT: upper branch, middle, lower branch, nerve trunk of facial nerve, orbicularis oris muscle, muscles of the nose, levators of the upper lip, and depressors of the lower lip. -ES: one electrode on the nerve trunk in front of the ear, while the other electrode was placed on the stimulated muscle (the frontalis and nasalis muscles)	- Group 1 (*n* = 15): low-level laser therapy (LLLT) - Group 2 (*n* = 15): ES - Group 3 (*n* = 15): control **Parameters**: Compound muscle action potentials (CMAPs) Latency of nerve action potential Sunnybrook facial grading system	**Adverse event**: not specified Short-term investigation revealed that LLLT was more efficient than ES in facial nerve regeneration for patients with Bell’s palsy. There was no statistically significant difference between Group 2 and 3.
Puls et al., 2020 [41]	Facial paralysis caused by surgical removal of benign or malignant tumor. Hypoglossal-facial-jump anastomosis (HFJA) in the last 12 years were selected	Retrospective study.	**Type**: transcutaneous electrodes **Duration**: biphasic triangular, 110 ms of pulse duration (twice per day for 10 min, 5 days per week) **Location**: zygomaticus muscle, depressor anguli oris muscle, and depressor labii muscle.	**Experiment 1** (HFJA) - Group 1: only surgery (*n* = 33) - Group 2: surgery + ES (*n* = 6) **Parameters**: rate of reinnervation by needle eletromyography **Experiment 2** (no HFJA) - Group 1: control (*n* = 7) - Group 2: ES (*n* = 6) **Parameters**: Sunnybrook score eFACE score	**Adverse event**: no No difference in time of reinnervation after facial nerve reconstruction surgery was observed between patients who did and did not receive ES. After spontaneous reinnervation, less synkinesis was noted. There was no evidence that ES prevents or delays reinnervation or increases synkinesis in facial paralysis.
Alakram et al., 2010 [15]	Bell’s palsy (less than 30 days post onset)	Prospective randomized study.	**Type**: transcutaneous electrodes **Duration**: a pulsed setting and frequency of 10 Hz, a pulse width/duration of 10 ms **Location**: frontalis or obicularis oculi, zygomaticus major (motor point 10 min, total 30 min)	- Group 1: control group, conventional therapy (*n* = 8) - Group 2: conventional therapy + transcutaneous electrical stimulation (TENS, *n* = 8) **Parameters**: Facial Disability Index	During the acute phase of Bell’s palsy, the clinical effects of electrical stimulation were noticeable but did not reach statistical significance.

**Abbreviations:** electrical stimulation, ES; low-level laser therapy, LLLT; Sub-threshold, continuous, low-frequency-impulse electrical stimulation, SCLES; transcutaneous electrical stimulation, TENS; compound muscle action potentials (CMAPs); Hypoglossal-facial-jump anastomosis (HFJA).

## Data Availability

Not applicable.
